# The value of narrow band imaging in diagnosis of head and neck cancer: a meta-analysis

**DOI:** 10.1038/s41598-017-19069-0

**Published:** 2018-01-11

**Authors:** Hui Zhou, Jing Zhang, Linghong Guo, Ji Nie, Chenjing Zhu, Xuelei Ma

**Affiliations:** 1 State Key Laboratory of Biotherapy and Cancer Center, West China Hospital, Sichuan University and Collaborative Innovation Center, Chengdu, PR China; 2 West China School of Medicine, West China Hospital, Sichuan University, Chengdu, PR China

## Abstract

Head and neck cancer is difficult to diagnose early. We aimed to estimate the diagnosis value of narrow band imaging(NBI) in head and neck cancers. We identified relevant studies through a search of PubMed, Embase and the Cochrane Library. We used a random effect model. Subgroup analysis and meta-regression analysis were performed to estimate the factors which may influence the sensitivity and specificity of the NBI. We included 25 studies with total 6187 lesions. The pooled sensitivity, specificity, positive likelihood rate, negative likelihood rate and diagnostic odds ratios of NBI were 88.5%, 95.6%, 12.33, 0.11 and 121.26, respectively. The overall area under the curve of SROC was 96.94%. The location, type of assessment, type of endoscope system and high definition were not significant sources of heterogeneity (*P* > 0.05). However, magnification may be related to the source of heterogeneity (*P* = 0.0065). Therefore, NBI may be a promising endoscopic tool in the diagnosis of head and neck cancer.

## Introduction

Head and neck cancer include oral cavity and oropharyngeal cancers, nasopharyngeal cancers (NPC), laryngeal and hypopharyngeal cancers, *et al*. According to the globocan 2012, the incidence of head and neck cancer every year is 683235/1000,000, the mortality is 375665/1000,000. And it is the sixth cause of cancer death throughout the world^1–3^. Because of the difficulty in early diagnosis, the five-year survival rate of head and neck cancer is lower than other cancers like breast, cervix and colorectal cancers^4^. Therefore, early diagnosis of head and neck cancer is extremely important.

There are many kinds of methods to detect head and neck cancer, including radiologic examinations and endoscopy. Radiologic examinations include CT, MRI, PET/CT and so on. CT or MRI provides cross sectional imaging, if the tumors are too small, they can’t be visualized by CT or MRI. The time and cost may be also problem for patients. Additionally, CT or MRI may be beneficial in staging laryngeal carcinoma and planning the most appropriate surgical procedure^5^. PET/CT identified primary tumors of non-squamous origin that infiltrated the middle or deep submucosal layer, including nonkeratinizing and undifferentiated carcinomas. Small or superficial lesions can easily be missed, because they are too small to take up sufficient FDG for detection on PET, this is the limited resolution of the PET scanner^6,7^. Therefore, endoscopy may be the prime method to early diagnose head and neck cancer. Among them, narrow band imaging (NBI) is a novel optical digital method of image-enhanced endoscopy which combined with the electronic laryngoscope. The thin capillary network on the mucosal surface shows as brownish and thick blood vessels show as cyan, on the NBI image. To identify suspicious lesions, especially in the colon, stomach, and esophagus, NBI has been applied as the standard endoscopic examination. Besides there were many meta-analysis studies to evaluate the diagnostic performance of NBI in these cancers^8–15^.

However, there was only one meta-analysis to systematically evaluate the value of NBI in diagnosing head and neck cancer^16^, to our knowledge, and there was another meta-analysis to assess the diagnostic performance of NBI for second primary lesions in patients with esophageal and head and neck cancer^17^. Therefore, we aimed to establish the sensitivity, specificity of NBI for differentiation between benign and malignant head and neck lesions.

## Results

### Study selection

**Figure. 1:**
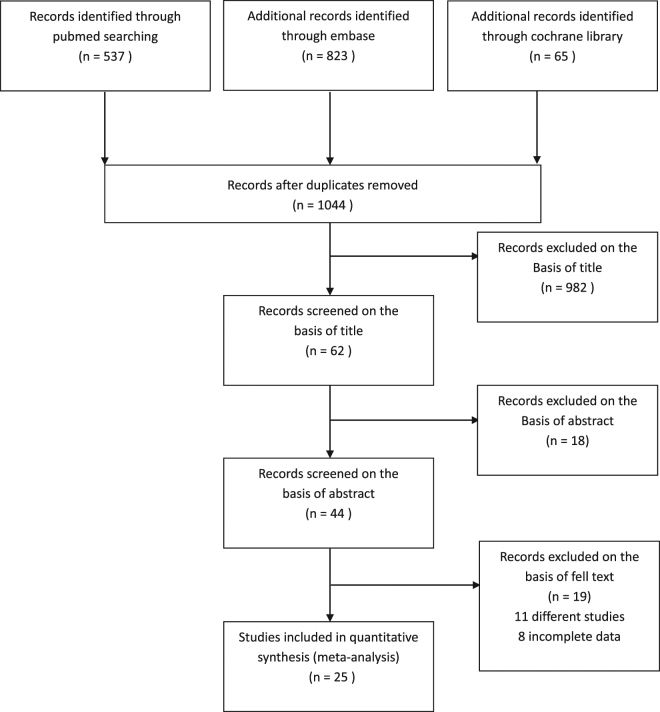
Study flow chart.

We included 25 studies with total 6187 lesions. The search process and articles eligible for this meta-analysis are shown in Fig. 1. From the initial keyword search, a total of 1044 studies after removing duplications were identified. After screening the title, sixty-two studies were left. Then, eighteen studies were excluded on the basis of abstract. Then, nineteen studies were excluded because of different studies (n = 11) and incomplete data (n = 8) after further reviewing in full text. Finally, twenty-five studies included in the meta-analysis, including two studies that had been reported in abstracts, twenty-three studies were full texts.

### Characteristics of eligible studies

The characteristics of the 25 included studies are summarized in Table 1^18–42^. All included studies were published in English except one which was published in Chinese^42^. The year of publication of studies ranged from 2008 to 2016. These studies were from 6 different countries, most of them were done in Asia Twenty (80%) studies were prospective, only five (20%) were retrospective. Four (16%) studies were performed with magnification system, other twenty-one (84%) studies were without magnification. And eight (32%) studies were assessed with high definition system, twenty (80%) studies were without it, three (12%) studies consisted of both high definition and no high definition. Six studies (24%) were done with Exera series endoscopy systems, three (12%) with Lucera spectrum endoscopy systems, and sixteen (64%) with unknown systems. Sensitivity for differentiation of lesions in all studies varied from 43.2% to 100%, and specificity from 74.5% to 100%. The numbers of patients and lesions, mean age the ratio of male and female, the numbers of pathologists and endoscopists also can be seen in the Table 1.

**Table 1:** Study characteristics of narrow band imaging.

	**Location**	**Author**	**Year**	**Country**	**Publication**	**Patients**	**Lesions**	**Mean Age**	**Male/Female**	**Evaluation**	**Pathologists**	**Endoscopists**	**Magnification**	**High Definition**	**Endoscopy system**	**Real Time**
1	nasopharyngeal carcinoma	Haidi Yang	2012	China	Full text	1854	1854	53.1 ± 8.3	1153/701	Prospective	2	—	no	no	—	real time
2	nasopharyngeal carcinoma	Ching YinHo	2011	China	Full text	63	63	46.8	46/17	Prospective	—	—	no	no	—	real time
3	nasopharyngeal carcinoma	Wen Hung Wang	2012	China	Full text	106	106	55.6 ± 11.9	80/26	Prospective	1	—	no	no	—	real time
4	nasopharyngeal carcinoma	Wen Hung Wang	2011	China	Full text	79	79	52.9 ± 21.8	58/21	Prospective	1	2	no	no	—	real time
5	nasopharyngeal carcinoma	Yi Hui Wen	2012	China	Full text	211	285	38	133/78	Prospective	2	2	no	no	Visera	real time
6	cancers in head and neck	Manabu Muto	2010	Japan	Full text	320	29	64	141/17	RCT	4	—	yes	no	Lucera	real time
7	cancers in head and neck	PhanNguyen	2013	Australia	Full text	73	43	64.15 ± 8.23	58/15	Prospective	—	2	no	no	—	real time
8	cancers in head and neck	CesarePiazza	2010	Italy	Full text	59	59	62	49/10	Prospective	1	3	no	yes	Exera	real time
9	cancers in head and neck	Akihito Watanabe	2008	Japan	Full text	667	667	65.9 ± 8.3	569/98	Prospective	1	—	no	no	—	real time
10	cancers in head and neck	Piazza C.	2011	Italy	Abstract	551	551	—	—	Prospective	—	—	no	yes	—	real time
11	oral and oropharyngeal cancers	GiancarloTirelli	2015	Italy	Full text	16	32	64.3	11/5	Prospective	1	2	no	no	Visera	real time
12	oral and oropharyngeal cancers	Shih Wei Yang	2015	China	Full text	72	72	54.6 ± 11.2	66/6	Retrospective	2	2	no	no	—	retrospect
13	oral and oropharyngeal cancers	AN Vu	2015	Australia	Full text	98	93	male:58.58 ± 9.42;female:62.09 ± 11.12	43/52	Prospective	1	1	yes	yes	Exera	real time
14	oral and oropharyngeal cancers	Shih Wei Yang	2014	China	Full text	63	63	57.9 ± 16.7	41/22	Retrospective	2	2	no	no	—	retrospect
15	oral and oropharyngeal cancers	S.-W. Yang	2013	China	Full text	317	317	52.4 ± 10.7	274/43	Retrospective	2	—	no	no	—	retrospect
16	oral and oropharyngeal cancers	S.-W. Yang	2012	China	Full text	414	414	52.15 ± 10.75	365/49	Retrospective	2	—	no	no	—	retrospect
17	oral and oropharyngeal cancers	Majorana A.	2010	Italy	Abstract	80	80	—	—	Prospective	—	—	no	yes	—	real time
18	laryngeal and hypopharyngeal cancers	Michal Zabrodsky	2014	Czech Republic	Full text	66	66	63	44/12	Prospective	—	3	yes	yes	Exera	real time
19	laryngeal and hypopharyngeal cancers	Giulia Bertino	2015	Italy	Full text	217	248	63.8	198/19	Retrospective	—	—	no	no	Exera	retrospect
20	laryngeal and hypopharyngeal cancers	X-G NI	2011	China	Full text	85	104	55	80/5	Prospective	2	1	no	no	Lucera	real time
21	laryngeal and hypopharyngeal cancers	Cesare Piazza	2010	Italy	Full text	279	279	65	253/26	Prospective	1	3	no	yes	Exera	real time
22	laryngeal and hypopharyngeal cancers	Akihito Watanabe	2009	Japan	Full text	34	35	69.5 ± 9.8	31/3	Prospective	—	—	no	no	—	real time
23	laryngeal and hypopharyngeal cancers	Hitomi Minami	2012	Japan	Full text	222	294	67.0 ± 16.0	169/53	Prospective	—	2	yes	yes	—	real time
24	laryngeal and hypopharyngeal cancers	Marcel Kraft	2016	Switzerland	Full text	205	205	60	153/52	Prospective	1	3	no	yes	Exera	real time
25	laryngeal and hypopharyngeal cancers	X-G NI	2010	China	Full text	122	149	56	113/9	Prospective	2	2	no	no	Lucera	real time

### The potential heterogeneity

Analyzed by meta-regression, the location, type of assessment, type of endoscope system and high definition were not significant sources of heterogeneity (*P* > 0.05). However, magnification may be related to the source of heterogeneity, diagnostic accuracy of NBI with magnification was 0.1 times higher than NBI without magnification (RDOR = 0.10, 95% CI: 0.02–0.49; *P* = 0.0065).

### The overall diagnostic accuracy of NBI

We pooled the sample statistics directly with Spearman correlation coefficient: −0.155, *p*-value = 0.461, which manifested there was no threshold effect. In terms of heterogeneity, we used a random effects model.

**Figure 2:**
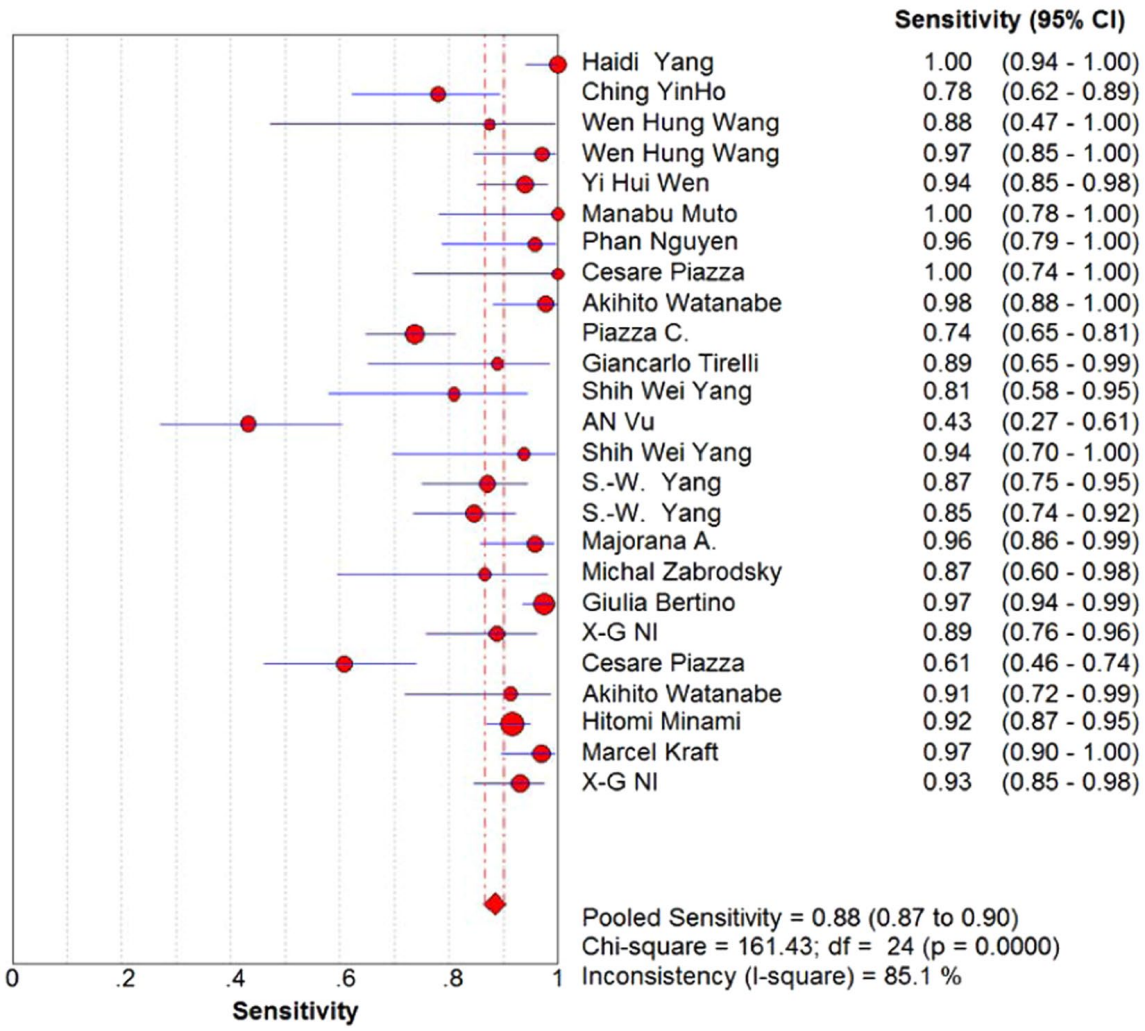
Forest plot showing pooled sensitivity of narrow-band imaging for head and neck cancer.

**Figure 3:**
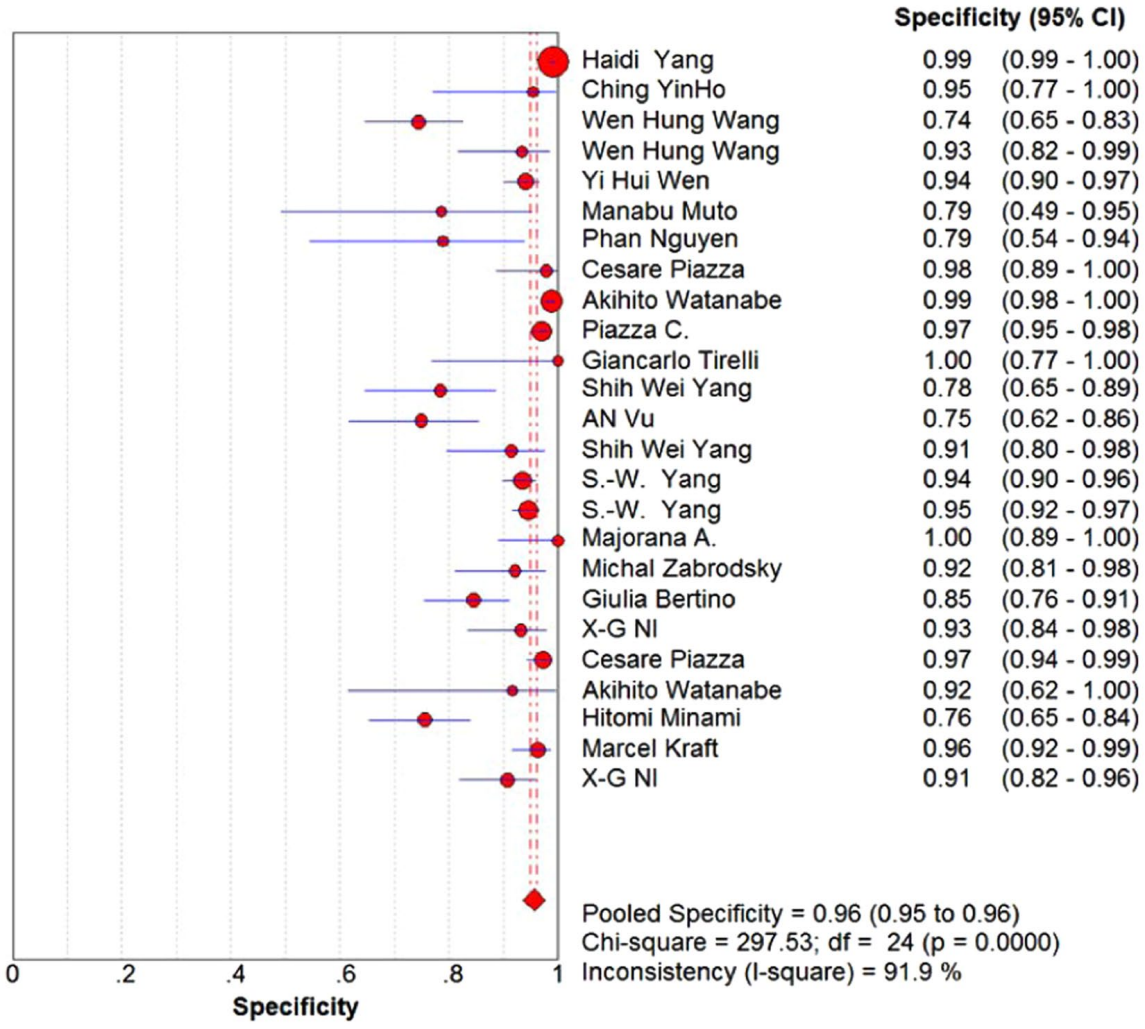
Forest plot showing pooled specificity of narrow-band imaging for head and neck cancer.

**Figure 4:**
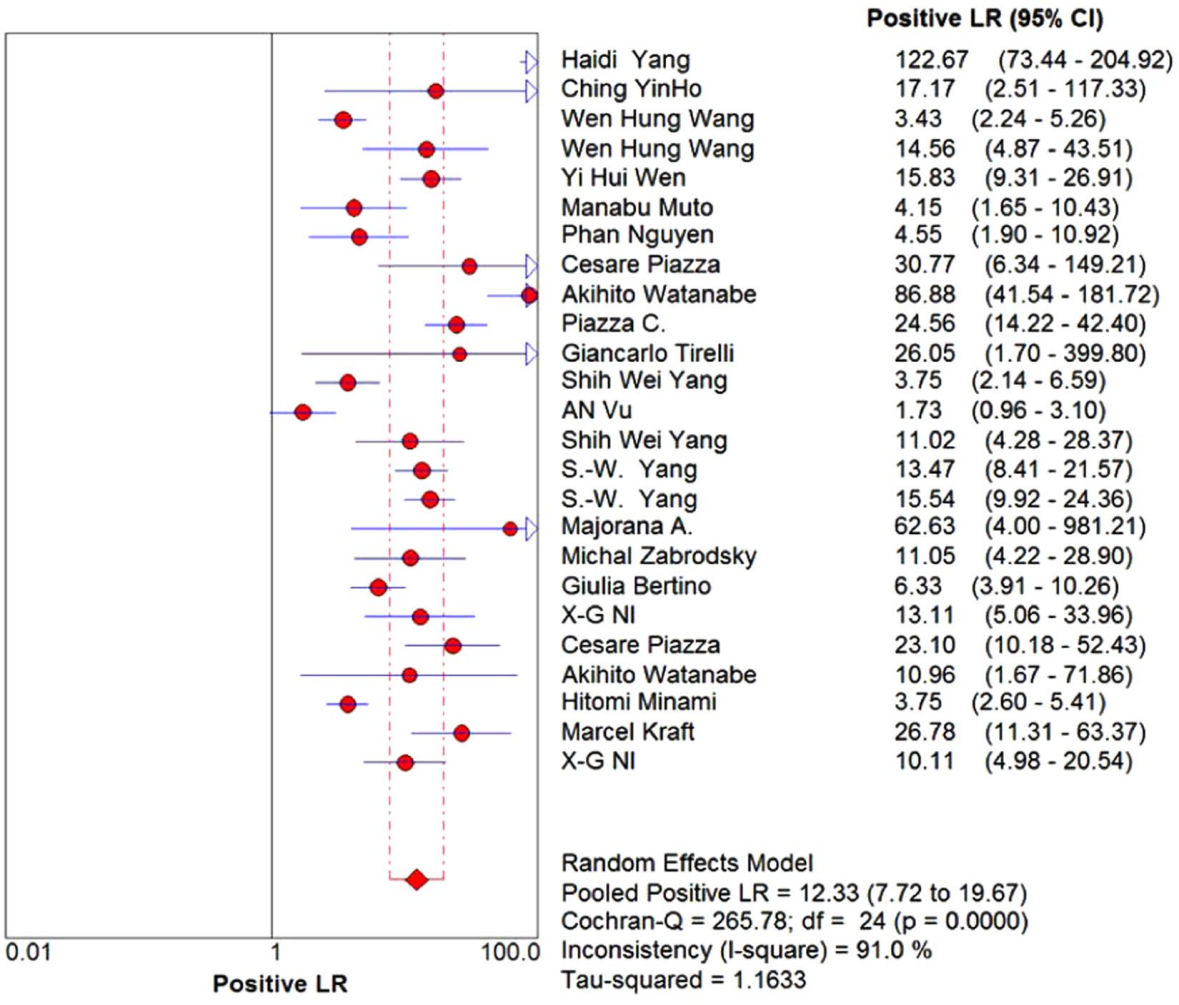
Forest plot showing pooled positive likelihood ratio of narrow-band imaging for head and neck cancer.

**Figure 5:**
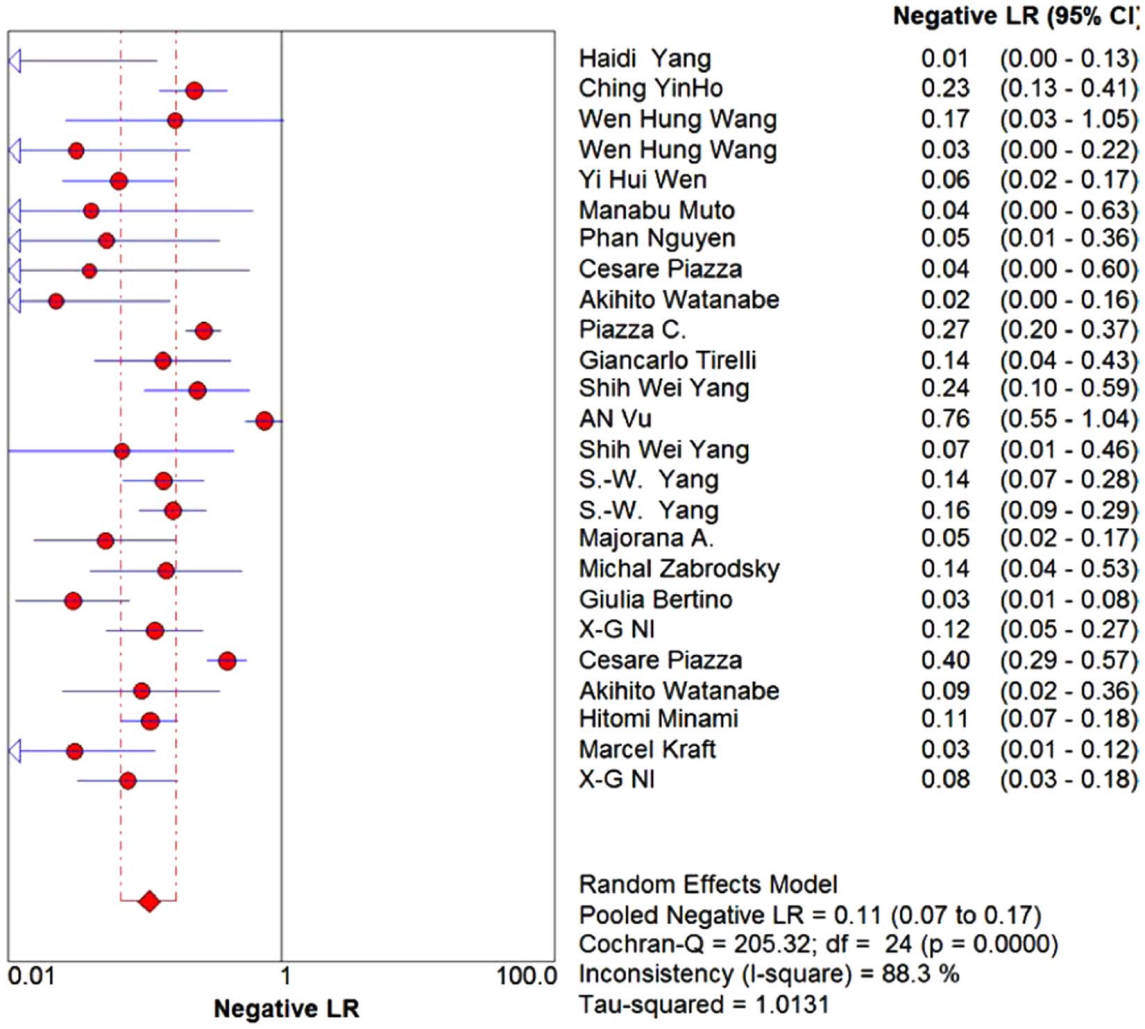
Forest plot showing pooled negative likelihood ratio of narrow-band imaging for head and neck cancer.

A total of 6187 lesions were detected. The pooled sensitivity and specificity of NBI were 88.5% (95%CI: 86.6–90.2, I^2^ = 85.1%, Figs. 2) and 95.6% (95%CI: 95.0–96.2, I^2^ = 91.9%, Fig. 3), respectively. The pooled PLR and NLR of NBI were 12.33 (95%CI: 7.72–19.67, I^2^ = 91%, Figs. 4) and 0.11 (95%CI: 0.07–0.17, I^2^ = 88.3%, Fig. 5), respectively. The pooled DOR was 121.26 (95%: 63.50–231.56, I^2^ = 80.8%, Supplementary Figure 1). The overall area under the curve (AUC) of SROC was 96.94%, Q statistic was 91.98%. (Supplementary Figure 2).

### The diagnostic accuracy of NBI in different locations

A total of 1349 lesions in the head and neck, 2387 lesions in nasopharyngeal carcinoma, 1071 lesions in oral and oropharyngeal cancers and 1380 lesions in laryngeal and hypopharyngeal cancers were detected. The sensitivity in nasopharyngeal carcinoma, laryngeal and hypopharyngeal cancers were higher than in the head and neck cancers, oral and oropharyngeal cancers (92.9% and 91.0% vs 84.6% and 81.9%). The specificity in head and neck cancers, nasopharyngeal carcinoma were higher than in oral and oropharyngeal cancers, laryngeal and hypopharyngeal cancers (97.5% and 97.4% vs 92.0% and 91.5%). Besides, the PLR of NBI in head and neck cancers, nasopharyngeal carcinoma were higher than in oral and oropharyngeal cancers, laryngeal and hypopharyngeal cancers. The NLR in head and neck cancers, nasopharyngeal carcinoma were lower than in oral and oropharyngeal cancers, laryngeal and hypopharyngeal cancers. The DOR and SROC also can be seen in Table 2.

**Table 2:** Diagnostic performance of narrow band imaging.

	Number of studies (lesions examined)	sensitivity (CI %,I^2^)	specificity (CI %,I^2^)	Positive LR (CI,I^2^)	Negative LR (CI,I^2^)	DOR (CI,I^2^)	SROC			
							AUC	SE (AUC)	Q^*^	SE (Q*)
Overall	25 (6187)	88.5% (86.6–90.2,85.1%)	95.6% (95.0–96.2,91.9%)	12.33 (7.72–19.67, 91%)	0.11 (0.07–0.17,88.3%)	121.26 (63.50–231.56,80.8%)	96.94%	0.82%	91.98%	1.32%
Cancers in head and neck	5 (1349)	84.6% (79.0–89.1,86.8%)	97.5% (96.5–98.4,84.2%)	16.47 (5.14–52.7, 89.6%)	0.07 (0.01–0.31,76.4%)	268.53 (54.09–1333.24,67.4%)	98.29%	1.26%	94.28%	2.51%
Nasopharyngeal carcinoma	5 (2387)	92.9% (88.5–96.0,79.8%)	97.4% (96.7–98.1,96.6%)	17.58 (3.68–84.04, 96.5%)	0.07 (0.02–0.31,84.6%)	260.41 (46.16–1469.12,72.9%)	98.32%	1.59%	94.34%	3.19%
Oral and oropharyngeal cancers	7 (1071)	81.9% (76.6–86.4,85%)	92.0% (89.9–93.8,83.3%)	8.47 (3.75–19.17, 88.2%)	0.17 (0.06–0.45,91.9%)	57.31 (12.29–267.15,89.4%)	94.53%	4.91%	88.42%	6.41%
Laryngeal and hypopharyngeal cancers	8 (1380)	91.0% (88.5–93.1,85.9%)	91.5% (89.3–93.4,84.1%)	10.49 (6.00–18.32, 78.7%)	0.10 (0.04–0.21,88.1%)	107.71 (52.41–221.36,61.3%)	96.55%	0.98%	91.27%	1.51%
Real Time	20 (5073)	87.5% (85.3–89.5,86.5%)	96.4% (95.8–96.9,92.4%)	13.75 (7.45–25.38,92.3%)	0.11 (0.06–0.18,89.7%)	142.22 (62.14–325.50,83.4%)	97.31%	0.93%	92.49%	1.57%
Post-procedure	5 (1114)	91.7% (88.1–94.5,75.8%)	91.9% (89.8–93.7,78.8%)	8.86 (5.12–15.32, 80.2%)	0.11 (0.06–0.22,66.4%)	84.12 (37.43–189.07,58.8%)	95.79%	1.52%	90.15%	2.18%
Magnification	4 (482)	86.0% (81.3–89.9,94.2%)	79.6% (73.5–84.8,61.0%)	3.91 (2.03–7.54,76%)	0.14 (0.02–0.81,95.5%)	28.49 (3.59–225.96,89.4%)	82.61%	2.82%	75.91%	2.56%
No Magnification	21 (5705)	89.3% (87.3–91.1,81.9%)	96.4% (95.8–96.9,90.6%)	15.55 (9.56–25.28, 89.1%)	0.10 (0.06-0.16,81.1%)	156.48 (92.00–266.14,60.6%)	97.56%	0.59%	92.93%	1.03%
High Definition	8 (1627)	91.6% (88.9–93.8,90.9%)	93.8% (92.2–95.1,90.2%)	13.50 (5.23–34.83, 92.8%)	0.06 (0.01–0.30,96.3%)	247.47 (34.02–1799.86,92.8%)	97.99%	1.47%	93.69%	2.75%
Not high definition	20 (5446)	88.6% (86.4–90.6,81.9%)	96.3% (95.7–96.8,91.2%)	13.73 (8.35–22.56, 89.8%)	0.11 (0.07–0.17,79.4%)	132.26 (79.30–20.60,55.7%)	97.17%	0.62%	92.25%	1.03%
Lucera	3 (282)	92.5% (86.6–96.3,36.1%)	90.6% (84.7–94.8,14.4%)	8.32 (4.34–15.97, 42.9%)	0.09 (0.05–0.16,0%)	121.27 (51.03–288.23,0%)	96.80%	1.08%	91.67%	1.71%
Exera	6 (950)	85.5% (81.3–89.1,94.7%)	92.8% (90.5–94.7, 87.2%)	10.65 (4.12–27.52, 88.9%)	0.13 (0.04–0.45,95.3%)	91.79 (12.67–665.19,92.3%)	96.88%	2.28%	91.79%	3.64%

### The diagnostic accuracy of NBI in different types of assessment

A total of 5073 lesions with real-time assessment and 1114 lesions with post-procedure were detected in retrospective assessment. The sensitivity with real-time was lower than post-procedure (81.5% vs 91.7%). The specificity was higher in real-time (96.4% vs 91.9%). The PLR of NBI in real time was higher than in post-procedure. The NLR, DOR and SROC also can be seen in Table 2.

### The diagnostic accuracy of NBI with or without magnification

A total of 482 lesions with magnification and 5705 lesions without magnification were detected. The sensitivity and specificity with magnification were both lower than without magnification (86.0% vs 89.3%, 79.6% vs 96.4%). The PLR of NBI with magnification was lower than without magnification. The NLR, DOR and SROC also can be seen in Table 2.

### The diagnostic accuracy of NBI with or without high definition

Three studies used both high definition technology and no high definition technology. A total of 1627 lesions with high definition and 5446 lesions without high definition were detected. The sensitivity with high definition was higher than without high definition (91.6% vs 88.6%). However, the specificity with high definition was lower than without high definition (93.8% vs 96.3%). The NLR of NBI with high definition was lower than without high definition. The PLR, DOR and SROC also can be seen in Table 2.

### The diagnostic accuracy of NBI in different types of endoscope system

A total of 282 lesions were detected by the type of Lucera and 950 lesions were detected by the type of Exera. The sensitivity in Lucera system was higher than in Exera system (92.5% vs 85.5%). However, the specificity in Lucera system was lower than in Exera (90.6% vs 92.8%). The NLR of NBI in Lucera was lower than in Exera. The PLR, DOR and SROC also can be seen in Table 2.

### Quality assessment and publication bias

Based on QUADAS, the quality assessment of included studies is presented in the Supplementary Table 1. A total of 17 studies scored more than 11 in 23 studies with full text. Besides, the first and fifth items scored more “no” than others. All of included studies scored “yes” in the third, sixth and seventh items. (Supplementary Table 2) There was no significant asymmetry in Deeks’ funnel plot (*P* = 0.447), representing no striking publication bias.

## Discussion

We analyzed the overall diagnostic accuracy of NBI, and found that NBI could be a considerable tool for differentiating benign and malignant lesions in head and neck cancer (sensitivity: 88.5%, specificity: 95.6%, PLR:12.33, NLR: 0.11). Additionally, we found that diagnostic accuracy of NBI was similar in the location, type of assessment, type of endoscope system and high definition (*P* > 0.05). However, diagnostic accuracy of NBI was different in magnification (*P* = 0.0065).

To the best of our knowledge, Cosway, B. *et al*. found that combined use of NBI and white light imaging (WLI) showed high diagnostic accuracies for primary, recurrent, and nasopharyngeal lesions^16^. This article only included 17 studies and emphasized more on the identification of primary tumors, recurrent tumors, cancers of unknown primary(CUP), and nasopharyngeal tumors. This meta-analysis included 25 studies with 6187 lesions. Meanwhile, we paid more attention on the locations of lesions and the methods of using NBI. In addition, one previous study showed that the sensitivity of NBI in detecting second primary neoplasm of head and neck cancer in patients with esophagus cancer was 87%^17^, similar with the sensitivity in this study. However the previous meta-analysis included only 3 studies to evaluate diagnostic performance of NBI for detecting head and neck cancer.

There are many studies investigating the performance of NBI in colon, gastric and esophagus cancer, *et al*.^8–13^, but there is rare study to explore the diagnostic value of NBI in the head and neck cancer^16,17^. Meanwhile, the difficulty of observing lesions of different locations in head and neck is different, thus the diagnostic accuracy may be also different. However, in this study, we didn’t find the difference. The sensitivity and specificity were both high. Additionally, the PLR of NBI were all more than 10 and the NLR were all less than 0.1 except in oral and oropharynx. Besides, the AUC of SROC were all more than 90%. The results indicated that NBI is a considerable tool to differentiate between benign and malignant lesions in head and neck cancer.

The type of included studies were RCT, prospective and retrospective, and we considered RCT and prospective as real time, the retrospective as post-procedure. Real-time assessment is the optimum selection to evaluate performance, because it avoids bias of photographic selection and performs an *in-vivo* optical diagnosis. However, real time assessment sometimes can not acquire the timely histological examinations. Thus, we analyzed the type of assessment to investigate the difference. The PLR and AUC of NBI in real time were higher than in post-procedure. However, we didn’t find the difference, which was consistent with the results of the previous study^8^. The small number of included studies may lead to this result. Therefore, a meta-analysis including more studies is needed to explain the reason.

With the development of optical technology, NBI can detect lesions with magnification or high definition system. Thus, this may influence the sensitivity and specificity of NBI. The utility of NBI is enhanced when it is employed with a magnifying endoscope^43,44^. This meta-analysis showed that NBI with magnification had a lower specificity (79.6%) than NBI without magnification (96.4%). Besides, the PLR and AUC of NBI with magnification were lower than without magnification. The results were not consistent with the previous study which indicated no significant difference between NBI with or without magnification^14,45,46^. This may because that the number of included studies of with or without magnification is extremely different. Additionally, the location is also different between this study which is in the head and neck and the previous studies which are in esophagus, stomach or colon. There are more polyps in stomach or colon cancer, while there is more squamous cell carcinoma in head and neck cancer. One study reported that high definition endoscopy could improve the detection and diagnosis of early gastric cancer^47^. However, this study showed that there was no difference between NBI with or without high definition. This is consistent with the previous study^48^. It may be related to the small number of studies including NBI with high definition. One previous study found that high definition significantly decreased the performance of NBI^12^. This previous study reported that this may because of the lack of experiences of endoscopists. More studies need be performed to explore the accuracy of NBI with or without high definition.

Different types of endoscope system may have different performance. The NBI technique developed by Olympus Medical Systems is now available in the most recent models of video-endoscopes that use the non-sequential system of illumination (Lucera Spectrum) or the sequential R/G/B system of illumination (Exera II). The two systems use different technology and improvements over time in the successive versions series endoscopes. Result in this study didn’t show the difference between the two systems, which is consistent with the previous study^49^. This indicated that the LUCERA and EXERA series provided the same clinical benefit in detecting head and neck cancer.

There were several limitations in this study. First, we didn’t know exactly the standard of experts and non-experts. In some studies, experts were experienced persons, and in other studies, experts were got trained persons, and another studies had no clear explanation of expert. Uneven standard greatly reduced the generalizability. Second, the diagnostic criteria were different. In some studies, they regarded a well-demarcated brownish area with scattered brown spots as susceptible lesions. Some studies used Ni’s classification, the classification of the microvascular endoscopic patterns of Ni, *et al*.^37^. Additionally, some studies used visualize intrapapillary capillary loops (IPCLs) pattern and a color change in the area between IPCLs. Several different classification systems used in the included studies also can reduce the generalizability of the overall performance. Because of the limitation of the numbers, we didn’t analyze this factor. Third, some studies included patients after treatment, while some studies included patients without treatment. This may influence the morphology of lesions, and influence the diagnosis Fourth, the heterogeneity of this study was relatively high. We showed that the NBI with or without magnification was the possible source. However, the time of detecting, the lesion size, the sample size, the pathological type and the bias of selection of patients can also reduce the generalizability of the overall performance and increase the heterogeneity of this study. Finally, our study only included studies written by English or Chinese.

In conclusion, narrow band imaging could be used by appropriately trained endoscopists to make a reliable optical diagnosis for head and neck lesions in daily practice. The location, type of assessment, type of endoscope system and high definition were not significant sources of heterogeneity (*P* > 0.05). The NBI with magnification should have better diagnosis accuracy. However, our article was not consistent with the hypothesis. More articles are needed to estimate the diagnosis value of NBI. Moreover, further studies should be focused on the diagnostic criteria, whether real-time and whether training will help improve the diagnostic performance of narrow band imaging.

## Methods

### Search strategy

We did a meta-analysis based on the Preferred Reporting Items for Systematic reviews and Meta-Analyses (PRISMA) guidelines^50^. We searched PubMed from inception to Feb 29, 2016, Embase from Jan 1, 1974 to Feb 29, 2016, Cochrane Library from Jan 1, 1999 to Feb 29, 2016 with following search terms: “narrow band imaging” or “NBI” and “sensitivity”. There was no restriction in PubMed and Embase. The studies were limited to trails in Cochrane Library. Additionally, we checked the reference lists of applicable studies to include other potentially eligible studies.

### Inclusion and exclusion criteria

In accordance with PICOS (participants, intervention, control, outcome, study design), the inclusion criteria were as follows:

1. Studies were in English or Chinese.
2. Studies included populations who were suspected to have oral cancers, oropharyngeal cancers, nasopharyngeal cancers, hypopharyngeal cancers, laryngeal cancers or head and neck cancers.
3. Studies investigated detection of narrow band imaging.
4. Studies took pathology as gold standard.
5. Studies provided data of true-positive (TP), false-positive (FP), true-negative (TN), and false-negative (FN); or reported data of sensitivity, specificity, positive predictive value (PPV), negative predictive value (NPV), and numbers of patients or lesions.
6. Studies were prospective, randomized controlled trails (RCTs) or retrospective.

The abstracts were also included when they contained available data.

The exclusion criteria were as follows:

1. Studies were in other languages.
2. Studies focused on other lesions, such as oral leukoplakia, ulcers.
3. Studies didn’t use narrow band imaging or had no pathological results.
4. Studies did not have available data.
5. Studies were other research types, such as reviews, case reports, letters.

### Data extraction

Two authors independently extracted the following data: the name of first author, year of publication, country, publication (full text or abstract), number of patients, number of lesions, patients’ characteristics (mean year, sex ratio), characteristics of narrow band imaging (magnification, high definition, endoscopy system, real time), endoscopists, pathologists, outcome data (TP, FP, FN, TN or sensitivity, specificity, PPV, NPV), and study design (prospective, RCTs, or retrospective). Extracted data were imported to a standard Excel (Microsoft Office). If there were any disagreements, the two authors would discuss and reach a consensus.

### Qualitative assessment

We used the Quality Assessment of Diagnostic Accuracy Studies (QUADAS) tool to assess risk of bias, applicability and reporting quality^1–3^. Each of the 14 items included in the original QUADAS tool is rated as “yes”, “no”, or “unclear”, with “yes” representing a positive outcome. The majority of the items (items 3, 4, 5, 6, 7, 10, 11, 12 and 14) is related to bias, with only two items related to variability (items 1 and 2) and the quality of reporting (items 8, 9 and 13), respectively^1^. Two independent authors performed and crosschecked the quality assessment.

### Statistical analysis

We estimated the pooled sensitivity, specificity, positive likelihood ratio (PLR), negative likelihood ratio (NLR) and diagnostic OR with corresponding 95%CI by a fixed effect model (Mantel-Haenszel method) when homogeneity was fine or a random effect model (DerSimonian-Laird method) when heterogeneity was significant, to indicate the accuracy of NBI in the diagnosis of benign and malignant tumors^51^. Besides, the summary receiver operating characteristic (SROC) curve was constructed as described by Moses *et al*.^18^ to show the results. The areas under the curve (AUC) were calculated to assess the diagnostic accuracy of NBI for the benign and malignant tumors.

We calculated Spearman correlation coefficient to analyze diagnostic threshold. If there was no threshold effect, we pooled these sample statistics directly. If there was threshold effect, we employed fit SROC curve and calculated the AUC and Q statistic. Besides, we used the inconsistency index (I^2^) to manifest the percentage variability attributable to heterogeneity. If the I^2^ value >50%, it demonstrates substantial heterogeneity. To investigate the potential sources, we performed subgroup analysis and meta-regression analysis to assess the effects of different locations, type of assessment (real-time vs post-procedure), magnification, high definition, and type of endoscope system.

Publication bias was assessed by Deeks’ funnel plot asymmetry test, with *p* > 0.05 representing no significant publication bias^52^. All statistical analysis were performed by using Meta-DiSc statistical software version 1.4 and STATA version 12.0.

### Data availaility

All the data we get was from public sources.

## Electronic supplementary material

Supplementary Information
