# Supplementary material for: The value of narrow band imaging in diagnosis of head and neck cancer: a meta-analysis
Source: Sci Rep. 2018 Jan 11;8:515. doi: 10.1038/s41598-017-19069-0 (PMC5765024; doi:10.1038/s41598-017-19069-0)
Supplement: Supplementary file 1 — Supplementary Information [file 41598_2017_19069_MOESM1_ESM.pdf]

The value of narrow band imaging in diagnosis of head and neck cancer: a meta-analysis

Hui Zhou[1,2], Jing Zhang[3], Linghong Guo[3], Ji Nie[1,2] Chenjing Zhu[1,2], Xuelei Ma[1,2,*].

[1] Lab of pathology, State Key Lab of Biotherapy, West China Hospital, Sichuan University, Chengdu, PR China

[2]Cancer Center, West China Hospital, Sichuan University, Chengdu, PR China

[3] West China School of Medicine, West China Hospital, Sichuan University, Chengdu, PR China

Note: Hui Zhou, Jing Zhang, Xuelei Ma contributed equally to this work.

***Corresponding author:** Xuelei Ma, West China Hospital, No.37, Guoxue Alley, Chengdu 610041, PR China; Tel: +86-28-85475576; Fax: +86 28 85502796; E-mail: drmaxuelei@gmail.com

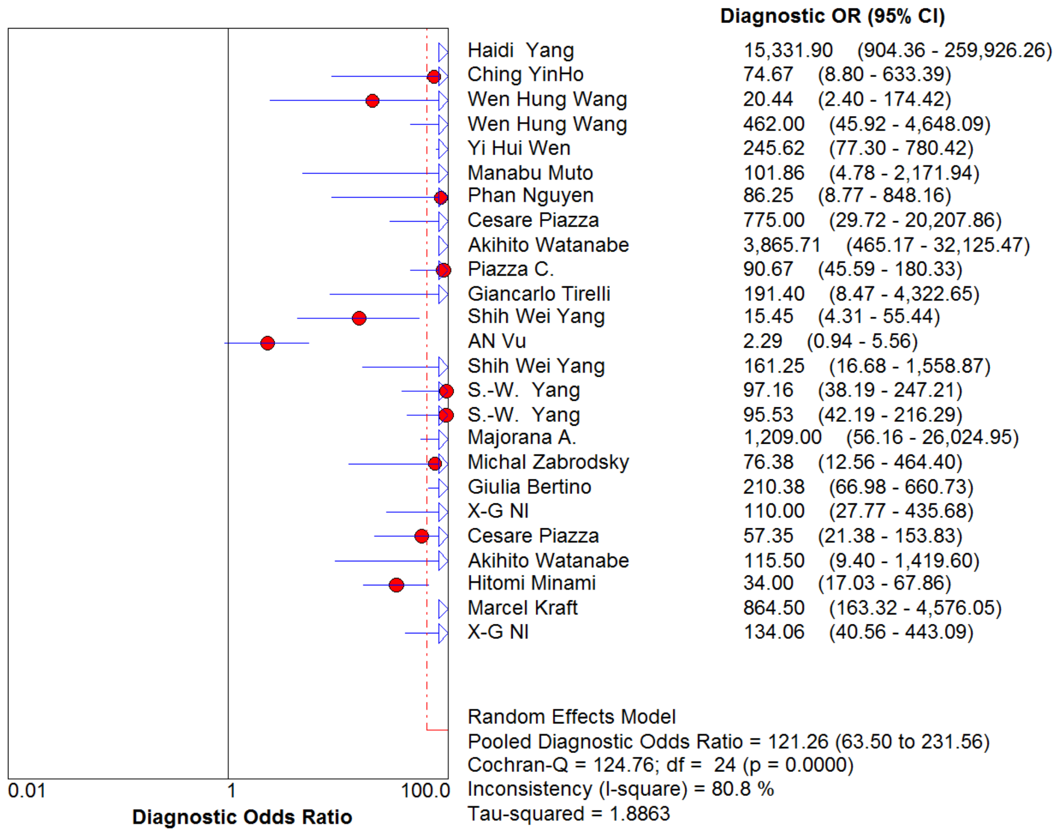

Supplementary figure 1. Forest plot showing diagnostic odds ratios of narrow-band imaging for head and neck cancer.

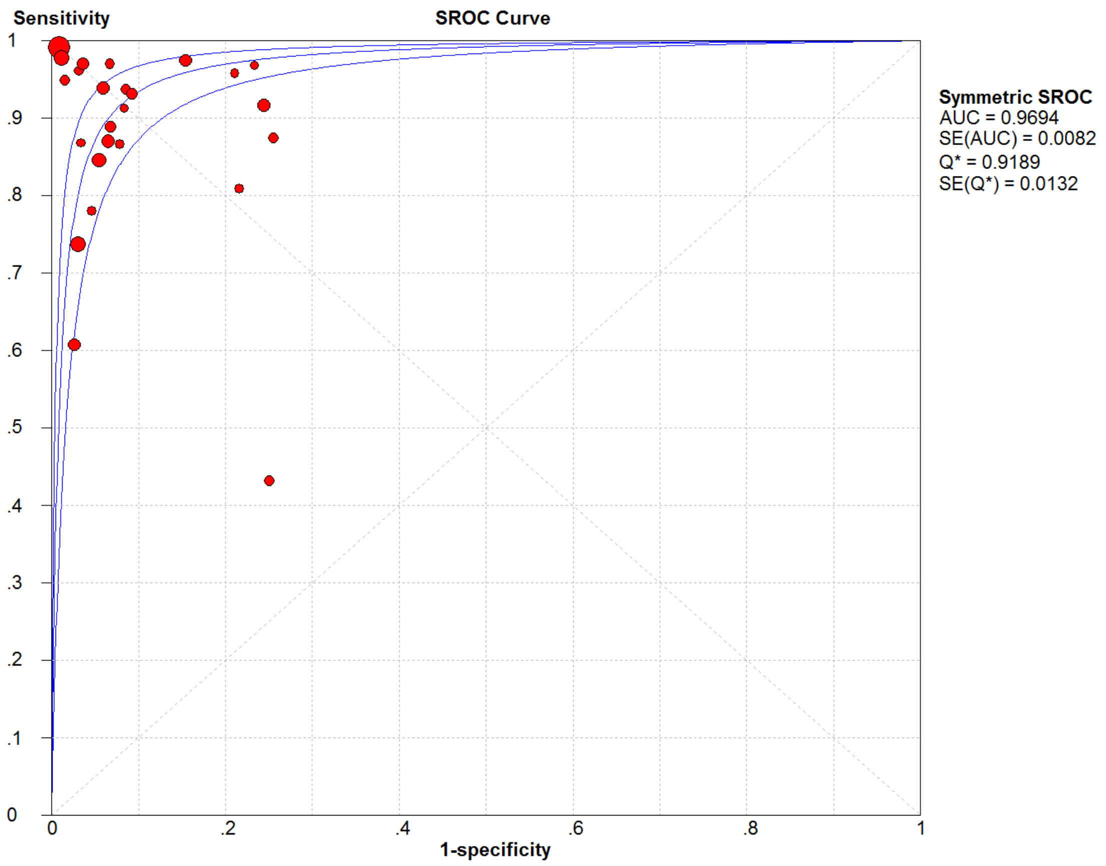

Supplementary figure 2. Summary receiver operating characteristic curve showing the diagnostic performance of narrow-band imaging for head and neck cancer

Supplementary stale 1. Quality assessment by QUADAS

included syudy	1	2	3	4	5	6	7	8	9	10	11	12	13	14	score
Haidi Yang,2012	+	-	+	+	-	+	+	+	-	-	+	+	+	-	9
Ching YinHo，2011	+	-	+	+	+	+	+	+	-	+	+	+	+	-	11
Wen Hung Wang,2012	+	+	+	+	-	+	+	+	-	+	+	+	+	+	12
Wen Hung Wang,2011	+	+	+	+	+	+	+	+	+	+	+	+	-	+	13
Yi-HuiWen,2012	+	+	+	+	+	+	+	+	+	+	+	+	+	+	14
Manabu Muto,2010	+	+	+	+	+	+	+	+	+	+	+	+	+	+	14
PhanNguyen,2013	+	+	+	+	+	+	+	+	-	+	+	+	-	+	12
CesarePiazza,2010	+	+	+	+	-	+	+	+	-	+	+	+	?	?	10
AkihitoWatanabe,2008	+	+	+	+	-	+	+	+	+	+	+	+	?	+	12
Piazza C.,2011	?	?	+	?	?	+	+	?	?	?	?	?	?	?	?
GiancarloTirelli,2015	+	+	+	+	-	+	+	+	-	-	+	?	?	+	9
Shih-WeiYang,2015	-	+	+	+	+	+	+	+	+	+	+	+	?	+	12
AN Vu,2015	+	+	+	+	+	+	+	+	+	+	+	+	+	+	14
Shih-WeiYang，2014	-	+	+	+	+	+	+	+	+	+	+	+	+	+	13
S.-W.　Yang,2013	-	+	+	+	+	+	+	+	+	+	+	?	+	+	12
S.-W.　Yang,2012	-	+	+	+	+	+	+	+	+	+	+	+	+	+	13
Majorana A.,2010	?	?	+	?	?	+	+	?	?	?	?	?	?	?	?
MichalZabrodsky,2014	-	+	+	-	-	+	+	+	-	+	-	+	+	+	9
GiuliaBertino,2015	-	+	+	?	-	+	+	+	-	+	-	+	+	+	9
X-G NI,2011	+	+	+	+	+	+	+	+	+	+	+	+	+	+	14
Cesare Piazza,2010	-	-	+	+	-	+	+	+	+	+	-	+	+	+	10
Akihito Watanabe,2009	+	-	+	+	+	+	+	+	-	+	+	+	+	+	12
HitomiMinami,2012	+	+	+	+	+	+	+	+	+	+	-	+	+	+	13
Marcel Kraft,2016	+	+	+	+	+	+	+	+	+	+	+	+	+	+	14
Ni XG,2010	+	+	+	+	+	+	+	+	+	+	+	+	+	+	14

supplementary table 2. The number and percentage of the score of each item

response	1	2	3	4	5	6	7	8	9	10	11	12	13	14
**yes**	16 (64%)	19 (76%)	25 (100%)	21 (84%)	15 (60%)	25 (100%)	25 (100%)	23 (92%)	14 (56%)	21 (84%)	19 (76%)	21 (84%)	17 (68%)	20 (80%)
**no**	7 (28%)	4 (16%)	0	1 (4%)	8 (32%)	0	0	0	9	2 (8%)	4 (16%)	0	2 (8%)	2 (8%)
**unclear**	2 (8%)	2 (8%)	0	3 (12%)	2 (8%)	0	0	2 (8%)	2	2 (8%)	2 (8%)	4 (16%)	6 (24%)	3 (12%)
